# The Alumni Associations and Their Influence for Good

**Published:** 1873-05

**Authors:** 


### ARTICLE VI.

### *The Alumni Associations and their Influences for Good.*

It is only within the past few years that the alumni of our different medical colleges have claimed for themselves any existence in fact. From originally small gatherings, these organizations have so increased that active members are scattered all over the country, and will travel scores of miles to attend the stated re-unions. There are many reasons why we should congratulate ourselves on such a state of things. Aside from the good which these meetings do to the members, individually and collectively, in affording opportunities for social enjoyment and professional encouragement, there is much more that can be done for the colleges, and through them for the educational interests of the profession at large. It must not be denied that the alumni associations are becoming foci of immense power and influence; that they are capable, by the very circumstances of their formation, of creating a healthy professional sentiment for reform which could not be done by any other means, They can in reality be the powers behind the throne, and, if they make proper use of their opportunities, can insist upon changes in the schools, which in the end will redound to the credit of all interested parties. The college must be poor indeed which, after an existence of a score of years, cannot count among its graduates men who have by sheer diligence risen to the front ranks of their profession—men who have obtained for themselves positions which, if their former teachers care not to envy, they must at least respect. These successful men are earnest in their endeavors to lessen these difficulties in their successors which their own hard-earned experiences have verified—they are actuated with the commendable disposition to prepare the future generations of medical men to combat these obstacles more successfully. In such a work they can afford to be independent of mere selfish considerations, with perhaps the single exception of a jealous pride in the success of their alma mater.

The alumni should always have a special influence with their alma mater on this very account. They must be presumed to be the best friends to their own college. There is no element but partiality towards an institution from which they have received their diplomas, and all the suggestion for reform should be so considered by the faculties. If the associations are outspoken in their convictions as to what is right, their opinions should receive all the weight which this acknowledgment of friendship to the governing powers should entitle them.

Already we have had evidences of their influence with respective teachers. On more than one occasion these very associations have taken up the discussion of matters pertaining to the colleges which even the faculties, from purely politic motives, did not dare to touch upon. They have borne the odium of adverse criticism with that independence which a conviction of being in the right alone can give, and have at last come off victorious, generously sharing the palm with a misguided but repentant faculty. Not only can the alumni of any institution step in at times to maintain a good name, but they are able and willing to increase the reputation and usefulness of these seats of learning. It is within their power to do for a college what it may in a pecuniary sense be incapable of doing for itself. In the shape of endowments, for instance, it can give a practical turn to the suggestions for reform in the shape of extra professorships, increased time for study, and the like, which assistance we can never expect to gain from any other quarter. In more than one of our colleges the initiative in this respect is already being taken with the promise of very gratifying results. This is one of the means to an end which gives us a pivot upon which to turn many of the time-worn suggestions of years past, and will give every one interested in educational matters a real and abiding interest in alumni associations. There are many other reasons why these organizations should prosper, but in our opinion this is the principal one, and if the alumni continue to take an interest in this matter the results cannot fail to satisfy the most sanguine. In order to smooth the way, however, we claim that the alumni association of each college should have a representation in the council: this is simple justice, and in many points of view the smallest possible compensation on the part of the faculties for the ultimate good to be attained.

The alumni associations of our colleges in this city are all in a prosperous condition—are, so to speak, permanent institutions. They number among their members some of the most influential men all over the country, men who know what is wanted in the way of reforms in medical education, and who are capable of backing up their convictions in a practical manner. We cannot believe that when the proper time comes for initiating the necessary changes any one of them will be found in the background.—*Med. Record.*

